# Retraction: Long noncoding RNA PCA3 regulates glycolysis, viability and apoptosis by mediating the miR-1/CDK4 axis in prostate cancer

**DOI:** 10.1039/d1ra90066h

**Published:** 2021-02-02

**Authors:** Laura Fisher

**Affiliations:** Royal Society of Chemistry Thomas Graham House, Science Park, Milton Road Cambridge CB4 0WF UK advances-rsc@rsc.org

## Abstract

Retraction of ‘Long noncoding RNA PCA3 regulates glycolysis, viability and apoptosis by mediating the miR-1/CDK4 axis in prostate cancer’ by Shuo Gu *et al.*, *RSC Adv.*, 2018, **8**, 37564–37572, DOI: 10.1039/C8RA08083F

The Royal Society of Chemistry hereby wholly retracts this *RSC Advances* article due to concerns with the reliability of the data. The images in the article were screened by an image integrity expert. The backgrounds to the western blot bands do not appear authentic and the blots may not be genuine. Furthermore, the blots and many other features of the article were found to closely resemble the blots and features of other articles, which is highly unexpected given that there are no overlapping authors.

The authors were asked to provide the raw data for this article, but did not respond. Given the significance of the concerns about the validity of the data, and the lack of raw data, the findings presented in this paper are not reliable.

The authors were informed but have not responded to any correspondence regarding the retraction.

Signed: Laura Fisher, Executive Editor, *RSC Advances*

Date: 19^th^ January 2021

## Supplementary Material

